# Are electric vehicle batteries being underused? A review of current practices and sources of circularity

**DOI:** 10.1016/j.jenvman.2023.117814

**Published:** 2023-07-15

**Authors:** Maite Etxandi-Santolaya, Lluc Canals Casals, Tomás Montes, Cristina Corchero

**Affiliations:** a Catalonia Institute for Energy Research (IREC), Energy Systems Analytics Group, Jardins de Les Dones de Negre 1, 2, 08930, Sant Adrià de Besòs, Barcelona, Spain; b Department of Engineering Projects and Construction, Universitat Politècnica de Catalunya-UPC, Jordi Girona 31, 08034, Barcelona, Spain

**Keywords:** Circular economy, Electric vehicle batteries, End of life, State of health

## Abstract

The increasing demand for Lithium-ion batteries for Electric Vehicle calls for the adoption of sustainable practices and a switch towards a circular economy-based system to ensure that the electrification of transportation does not come at a high environmental cost. While driving patterns have not changed much over the years, the current Electric Vehicle market is evolving towards models with higher battery capacities. In addition, these batteries are considered to reach the End of Life at 70–80% State of Health, regardless of their capacity and application requirements. These issues may cause an underuse of the batteries and, therefore, hinder the sustainability of the Electric Vehicle. The goal of this study is to review and compare the circular processes available around Electric Vehicle batteries. The review highlights the importance of prioritizing the first-life of the battery onboard, starting with reducing the nominal capacity of the models. In cases where the battery is in risk of reaching the End of Life with additional value, Vehicle to Grid is encouraged over the deployment of second-life applications, which are being strongly promoted through institutional fundings in Europe. As a result of the identified research gaps, the methodological framework for the estimation of a functional End of Life is proposed, which constitutes a valuable tool for sustainable decision-making and allows to identify a more accurate End of Life, rather than considering the fixed threshold assumed in the literature.

## Highlights

• Current EVs have the risk of being underused due to the high battery capacities.
• All batteries are assumed to be retired at 80% SoH regardless of the application.
• Circular practices during the first stages of the lifetime must be prioritized.
• A methodological framework for defining a functional EoL is provided.

## Graphical abstract

**Figure:**
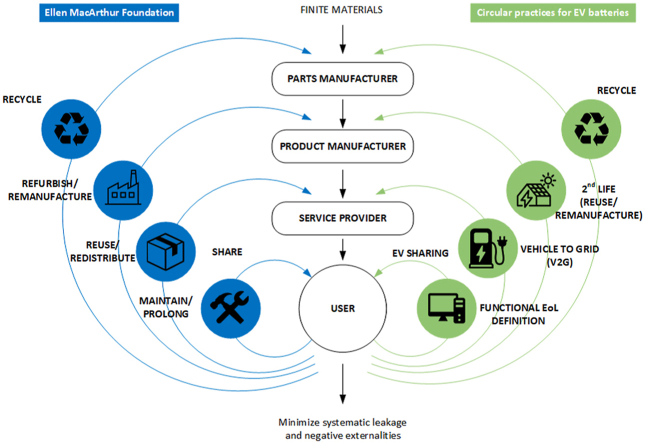

## 1 Introduction

The adoption of the Electric Vehicle (EV) is being promoted worldwide through sustained policy support with the aim of reducing the environmental impact of the transportation sector. After a decade of rapid growth, the number of EVs on the road has reached 16.5 million in 2021, triple the amount in 2018 (International Energy Agency (IEA), 2022). This value is expected to grow even faster in the upcoming years, as EVs take a central position in the market share (Lowell and Huntington, 2021).

At the heart of the current EV lies the Lithium-ion (Li-ion) battery. Li-ion batteries dominate the EV market over other types (Lead-acid, Lithium-Sulphur or Nickel Metal Hydride) due to their higher energy density, a crucial aspect in mobility, along with other benefits such as longer service lives (Sopha et al., 2022), high efficiencies (Stan et al., 2014) and a very limited self-discharge rate (Chen et al., 2012).

**Fig. 1:**
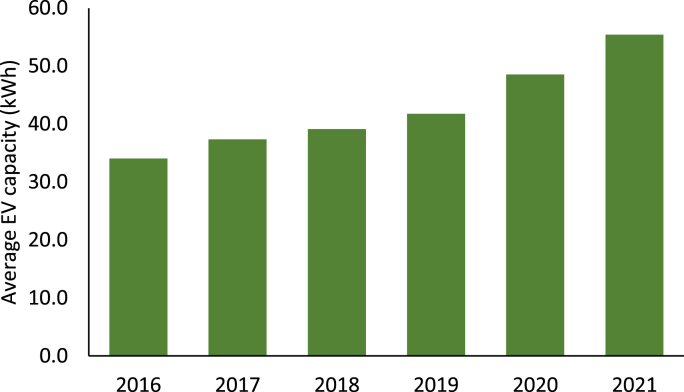
Evolution of the EV battery capacity over time (2016–2021).

Market trends show an important evolution over the years. Early EV models had batteries with 16 or 24 kW h of capacity. Considering the limited capacity, the low range was one of the biggest arguments against EV adoption, generating the so-called range anxiety (Riezenman, 1992) and, since then, battery capacities have experienced a constant increase over the years. Looking at the worldwide market, in 2021, the most sold EV was the Tesla Model Y with 75 kW h and higher capacity EVs keep launching on the market (e.g. Ford Mustang Mach-E with 98.8 kW h or Audi e-tron with 95 kW h). Fig. 1 shows the evolution of the average capacity of the EVs from the top 10 most-selling brands in the UK (UK Department for Transport, n.d.).

However, batteries represent an important part of the cost and carbon footprint of the vehicle (Bauer et al., 2015; Kawamoto et al., 2019). Moreover, besides the emissions related to the manufacturing of Li-ion batteries, some of the materials required in this process (Cobalt, Nickel, Manganese, Lithium and Copper) are considered scarce and their reserves are expected to be depleted in the not-so-far future with the current demand trends of the market (Simon et al., 2015; Weil et al., 2018).

This fast EV market growth, coupled with the increased capacity of recent EV models, raises fair concerns over the sustainability of EVs. The extensive use of resources for manufacturing large-capacity batteries should be backed by a need to satisfy the requirements imposed by EV drivers. However, considering that driving trips have not changed over recent years (Dalla Chiara et al., 2019), it is worth questioning whether the capacity increase is justified or whether it may lead to an underuse of the battery.

Considering the environmental issues mentioned, some voices suggest that other technologies will replace the battery EV as the central element in the transportation sector. Among EVs, other alternatives include Hybrid EVs (HEV) or Fuel Cell EVs (FCEV). A recent study compared, using different criteria, gasoline Internal Combustion Vehicles (ICVs) with battery EVs, FCEVs and HEVs (Cremades and Canals Casals, 2022) stating that only when environmental aspects gain weight, FCEVs and battery EVs are best positioned. According to the study, battery EVs seem to be a transitory alternative until FCEVs reach technological maturity. Alternative fuel ICVs (hydrogen or biofuels like methanol) are also investigated by researchers. A recent LCA analysed conventional ICVs, alternative-fuelled ICVs and EVs and showed that the hydrogen ICV was the most environmentally friendly option in all impact categories (Bicer and Dincer, 2018). Nevertheless, when compared to FCVs, hydrogen ICVs have lower efficiencies and a higher environmental impact (Acar and Dincer, 2020).

After all, whether the Li-ion battery EV is the most sustainable and adequate form of transportation remains under discussion. It is quite likely that not a single technology will dominate the market and that different ones will coexist (Wanitschke and Hoffmann, 2020). Nevertheless, it is clear that Li-ion EVs are going to hold an important market share in the upcoming years. Therefore, current trends should be reviewed to find gaps for improvement and, in this sense, a data-driven and circular economy-based treatment of the batteries should be pushed to make sure that the resources are used optimally and that batteries reach the recycling process with no residual value (Kurdve et al., 2019; Olsson et al., 2018).

When discussing sustainability practices in a growing market like that of EVs, the circular economy provides a valuable guideline. The circular economy is considered a solution for harmonizing economic growth and environmental protection by avoiding natural resource depletion and environmental degradation (Lieder and Rashid, 2016). The circular model challenges the dominant linear one of “take, make and dispose” by promoting actions to maintain the value of products, materials and resources in the economy for as long as possible while minimizing the generation of waste (European Commission, 2015).

The practices supported in this framework are related to closing, narrowing and slowing the resource loops (Bocken et al., 2016). Closing the loop refers to the recycling activities that minimize the use of new resources for manufacturing. Narrowing resource flows aims to use fewer resources per product and improve efficiency. Slowing the loop is related to the extension of the lifetime of a product, which defines its replacement speed and thus the consumption of natural resources required for their manufacture and the amount of waste they create (Stahel, 2013).

Previous reviews are available in the literature that have considered the circular economy framework applied to EV batteries. Four potential circular economy strategies were proposed in a recent work: reduction or elimination of Cobalt in the batteries, reuse and recycling (Baars et al., 2021). All strategies allowed reducing the demand for imported Cobalt in Europe. However, other environmental implications such as demand for other materials or Green House Gas (GHG) emissions were not considered in the study. A different work briefly reviewed each of the steps in the battery lifecycle, but then focused on recycling technologies and waste preparation and pre-treatment processes (Mossali, 2020). Other circular economy studies focus on comparing End of Life (EoL) processes (i.e. reuse vs recycling) of EV batteries (Ali et al., 2021; Pagliaro and Meneguzzo, 2019).

All of the referenced reviews miss important circular processes and focus only on a few of them, leaving aside other essential aspects like first-life usage maximization, which is what the present study focuses on. In fact, according to the circular economy, first-life-related actions (e.g. extending the use of the battery) should be the priority and not later stages like reusing, repurposing or recycling.

Consequently, this work aims to analyse the current use of the EV batteries and review the possible practices that can help to maximize the value of EV batteries. The main research questions addressed are.

• Which are the potential causes for current battery underuse?
• Which actions should be prioritized to avoid underuse and better use of resources?
• Is the battery End of Life correctly predicted? How should it be defined to provide a realistic estimation of health at retirement?

Therefore, the novelty of this study comes from: i) the potential underuse of the battery is analysed and highlighted as a major drawback to consider the EV as a sustainable form of transportation; ii) unlike existing reviews on circular economy applied to EV batteries, this review gives more weight to the first stages of the lifecycle; iii) Finally, as a result and key contribution, this study proposes a framework to fill an existing research gap related to providing an accurate estimation of the EoL.

This paper is structured as follows. Section 2 analyses the current usage of EV batteries to evaluate their potential underuse. The different circular economy-based alternatives are reviewed in Section 3. Section 4 reviews the state-of-the-art algorithms for the State of Health (SoH) estimation and Remaining Useful Life (RUL) prediction, highlighting their limitations. A framework to overcome these limitations is presented in Section 5 by presenting the State of Function (SoF) and its use to estimate a functional EoL. Section 6 presents the discussion and Section 7 finalizes the document presenting the conclusions extracted.

## 2 Underuse of the batteries: driving requirements among the entire first-life

This section aims to assess the actual driving requirements to evaluate whether batteries have the risk of being underused. Several publications have analysed the common driving distances of the European population. A recent study analysed data from more than one thousand vehicles over more than one year in different European countries (Dalla Chiara et al., 2019). In an urban context, the obtained results showed that 50% of the daily driving distances were below 5 km and 99.9% below 50 km. These values were significantly different for highway roads, where on 60% of the days less than 50 km were driven and less than 400 km for 99% of the days. A different study analysed values from German and Swedish datasets and concluded that for 75% of the days, the distance covered was 65 km and 72.3 km for Germany and Sweden respectively. (Plötz et al., 2017). This last study proposed that the daily driving distances can be represented using a Weibull distribution, according to which 50% of the population drives less than 34 km and 95% less than 89 km.

Finding the battery capacity requires translating the driving distances into energy consumption, for which different factors must be considered. The specific energy consumption of different EVs is commonly given by the measured consumption under the World Harmonized Light-duty Vehicle Test Procedure (WLTP) cycle at specific conditions. The authors have considered different EV models grouped by battery capacity to evaluate their consumption range. Other aspects, such as the ambient temperature or the driving style that will be discussed later in this work, increase the specific energy consumption of EVs. As a conservative estimation, an increase of 60% in consumption has been considered to account for high consumption cases (Laurikko et al., 2013).

**Fig. 2:**
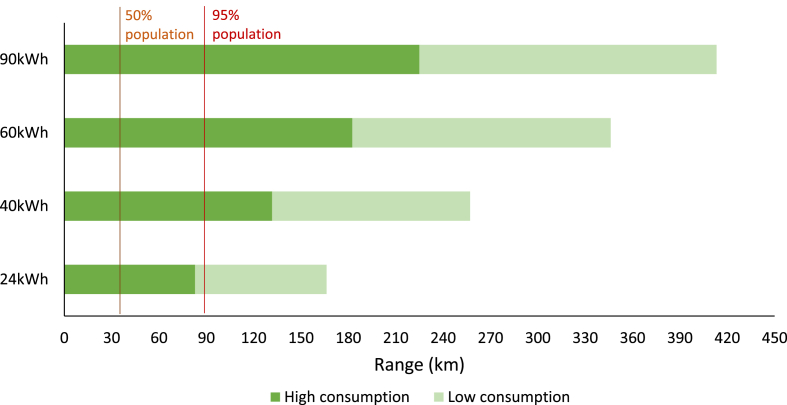
Estimated range for different battery capacities.

Considering the previous values and a useful battery capacity of 90% of the nominal one, which is the average of the EV models analysed, the driving ranges that different batteries can cover are presented in Fig. 2. The vertical lines correspond to the daily driving distances of 50% and 95% of the population according to the Weibull distribution referenced previously. It can be seen how the average daily distances, considering the high consumption case, can be covered with the smallest 24 kWh battery and the 95% case with the 40 kWh one.

A recent interview-based study showed that, even though some experts argue that to fully avoid range anxiety in users EVs should compete with diesel vehicles, others suggest that a 41 kWh could be enough to meet 95% of the driving requirements in a cold environment like Denmark, which is in line with Fig. 2 (Noel et al., 2020). A different study simulated driving trips from Switzerland and Finland and concluded that 90% and 85% of the driver needs in each country could be covered with existing EVs in 2018 (Melliger et al., 2018). According to Fig. 1, in 2018 the average battery capacity was between 30 and 40 kW h.

Therefore, it seems that battery capacities would not pose a barrier and that lower capacities than those appearing on the market nowadays are sufficient for the majority of the population in Europe. Consequently, the increase in battery capacity can lead to an important unused battery capacity in the Beginning of Life (BoL) for many drivers.

Nevertheless, the battery should not only be designed to meet the driving requirements at BoL but also to consider the entire lifetime of the EV until they reach the EoL. Several issues can lead to the end of the battery's first life. If the vehicle suffers from a crash, is damaged or has reached the EoL for any other reason, the EV battery must be taken out of the vehicle.

The battery might also reach the EoL when it is no longer able to provide the required functionalities for a specific driver, even before the EV has reached its EoL. Both as a consequence of time and driving, batteries suffer from an irreversible loss of functionalities known as degradation (Barré et al., 2013). The two main effects of battery degradation are the capacity fade and the internal resistance increase (Vetter et al., 2005).

The capacity fade directly reduces the distance that the EV can cover. The internal resistance is related to the power that the battery can provide or receive without reaching its operating limits and to the self-heating process (Zhuo Yang et al., 2017b). In terms of power, an increase in the internal resistance can reduce the ability of the EV to accelerate and drive uphill. The increased resistance also limits the maximum charging power that the battery can accept, which reduces regenerative braking capabilities and increases charging times (Saxena et al., 2015). In addition, a higher internal resistance increases the heat generation in the battery (Wang et al., 2018), following the Joules equation, which entails higher losses and reduced efficiency. The additional energy needed to cool the battery increases the energy consumption (Lu et al., 2010) and, if heat cannot be properly evacuated, safety issues may arise (Zhang et al., 2018).

Therefore, depending on the requirements imposed by the driver, after a certain level of degradation, the battery may not be safe to use or may not be able to provide the necessary range or power. At this point, the battery should reach EoL and be retired from the vehicle. However, this is not the case when looking at the research around EV batteries, as the literature defines the EoL assuming a universal SoH value for all cases.

Currently, the EoL criteria used for EV batteries defines that when the SoH falls to 70–80% the battery is no longer useful for automotive purposes (E. Martinez-Laserna et al., 2018a). This EoL threshold has been present since the development of the first low-capacity EV models. Setting the fixed threshold was done to avoid customers from noticing important losses of performance in their EV and it is useful when defining battery warranties.

The first authors to question the definition of the EoL threshold were Saxena et al. who analysed both capacity and power requirements based on an EV simulation (Saxena et al., 2015). Neither the capacity fade nor the internal resistance increase was seen as an important impediment for driving with batteries below the 70–80% SoH threshold. However, their analysis relied on a simulation and the use of standard driving cycles, which are not strictly representative of real stochastic patterns (Baure and Dubarry, 2019).

Later, another study on real driving trips from different European countries analysed statistically the energy requirements to fulfil the daily mobility needs (Canals Casals et al., 2019). It was concluded that lower values of SoH would still allow meeting the majority of the driving trips, which were seen to be on average 5 kWh and only occasionally exceeded 10 kWh. It was already highlighted that the increase of the internal resistance and the possible start of the ageing knee should be further analysed to validate the hypothesis of defining the EoL at higher levels of degradation.

The fixed EoL threshold is present in many research areas, such as those developing RUL algorithms (Catelani et al., 2021; Chen et al., 2022), which seems to be an oversimplification, considering that this threshold does not follow functional criteria.

In addition, studies that analyse the possibility of reusing EoL batteries are done taking as a premise that the retired batteries will still have an SoH of 70–80% (Bobba et al., 2019; Janota et al., 2020). In many cases, especially considering large battery capacities, the battery may still be functional at higher levels of degradation. Therefore, if their first life is pushed to this functional limit, the lifespan and the functionalities of the battery will be poorer, which directly affects the technical and economical assessment of battery reuse.

Therefore, two critical aspects have been highlighted that hinder the sustainability of EVs.

• Current battery capacities are, in many cases, significantly larger than the actual range needs of many drivers.
• A universal threshold for EoL is considered in the literature, which does not represent a functional value. Few studies suggest that the value of SoH at the EoL could be lower than the currently assumed one.

The increased capacity and the restrictive EoL threshold imply a potential underuse of the EV battery which calls for larger efforts into prioritizing strategies to avoid it. However, the lack of knowledge on the real SoH at the EoL caused by assuming a fixed threshold generates uncertainty around the assessment of what actions to promote. The following sections address these issues by first reviewing the existing circular practices that can be used to maximize the usage of the EV batteries and then by proposing a framework to estimate the functional EoL.

## 3 Alternatives for a sustainable treatment of EV batteries

**Fig. 3:**
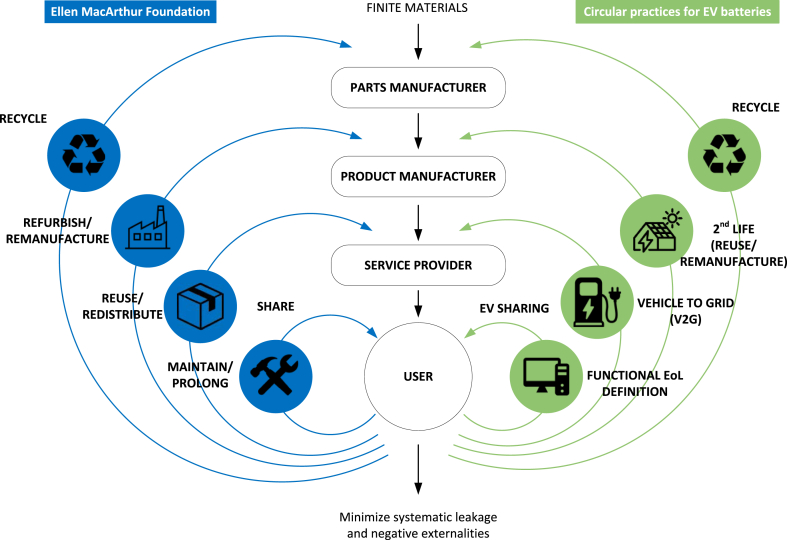
Butterfly diagram for EV batteries.

A common representation of the circular economy is provided by the butterfly diagram developed by the Ellen MacArthur Foundation. The butterfly diagram, represented on the left side of Fig. 3, shows the different technical cycles that aim to maintain the materials, components and products at their highest value at all times. The size of the circles represents the additional resource use that implementing each action entails.

Tighter circles are those with the lowest resource leakage and environmental impact (Ellen MacArthur Foundation, 2013). Therefore, the most effective cycle is to prolong the use of a product for as long as possible by maintaining the product in use without replacement or by repairing it. Similarly, the value of a product can be maintained by directly reusing it or redistributing it to a new user or sharing the product among different consumers. In this way, instead of manufacturing a new product, the one that already exists can serve several users or applications. If the product cannot be directly used, refurbishing or remanufacturing allows to maintain the value of the materials by building new products using the components and materials from the original one. Finally, if the product cannot be remanufactured, recycling allows to close the resource loop and minimize the extraction of new materials for the manufacturing processes.

Referring back to the butterfly diagram, the right side of Fig. 3 shows the different cycles for EVs, matching the theoretical ones, which will be reviewed later in this section. It should be highlighted that two of the cycles, namely Vehicle to Grid (V2G) and functional EoL definition, are not considered when discussing the circular economy and EV batteries and thus represent a novelty of this review.

A circular economy starts at the beginning of a product's life cycle through an adequate design and efficient production avoiding carbon-intensive energy sources. Therefore, before analysing the processes represented in Fig. 3, the first step is to look at the manufacturing of Li-ion batteries for EVs.

Better design can make products more durable or easier to repair, upgrade or remanufacture and can improve the disassembly process for recycling and reuse (European Commission, 2015). Standardization of the battery design at all levels (Beaudet et al., 2020), battery labelling (Gaines et al., 2018) and design for an easy opening and disassembly of the battery (Erythropel et al., 2018) are examples of current lines of improvements. These actions would enable the automation of the disassembly process, which is expected to improve the profitability of recycling and reduce the risk associated with EoL batteries (Harper et al., 2019).

An important part of EV battery research in the first stage of the battery lifecycle is led to developing new battery chemistries with more abundant materials, especially for the cathode composition (Fujita et al., 2021). High efforts are put into reducing the dependency on Cobalt, which is considered to be the most critical element both for environmental issues and for unethical sourcing practices (Harper et al., 2019). The dominant chemistries for the Li-ion cathode for EV batteries are NMC and LCO. However, Cobalt-free chemistries are expected to gain a high market share in the future. Besides Cobalt, Lithium which is used in all commercial chemistries is also considered critical (Weil et al., 2018) and its extraction requires the use of large amounts of water in regions where reserves are limited (Harper et al., 2019). Modifying the battery chemistries would reduce the supply risk of key materials while recycling processes advance.

However, regardless of the chemistry, a direct way of reducing the materials for manufacturing a single battery is to optimize its size. It has been discussed in Section 2 that most driving needs can be covered with lower capacities than those currently appearing in the market. The option of reducing the battery capacity can be a straightforward way of narrowing the resource loop and delaying the shortage of materials while reducing the weight of the EV, which improves its consumption. The minimum battery capacity needed should be further evaluated by an in-depth analysis of the driving needs of the population.

Meeting driving range needs with a low-capacity battery goes hand in hand with the development of other parallel actions (Shi et al., 2019). For most drivers, large trips are sporadic and only represent a small share of the total ones (Plötz and Sprei, 2021). Therefore, those trips could be accommodated using other resources instead of increasing the EV range, which would remain underused most of the time. One of those options is counting on a properly planned network of fast chargers along main driving routes (Bonges and Lusk, 2016). In this way, the users could perform a full charge of the battery making use of a stop that is made anyways for long-distance travel. Alternatively, instead of using the EV for long-distance travel, public transport or even renting an ICV could be considered.

Nevertheless, even if lower-capacity batteries could provide the functionalities required by most drivers, it is likely that market trends will not take this direction, as high autonomies are attractive to customers. Therefore, for those cases where the battery capacity may lead to an underuse of the battery, new circular processes should be pursued. Out of the ones represented in Fig. 3, recycling was the first concept to be developed and is often considered the pillar to improve the sustainability of the EV battery.

### 3.1 Recycling

One of the key concepts of the circular economy is closing the resource loop, for which recycling is the only alternative. Recycling is so relevant and has been so widely studied because it is a necessary stage that all EV batteries should reach no matter what they do before reaching this point. Recycling enables to decouple battery manufacturing from raw resource extraction. As discussed in the introduction, a shortage of key materials for battery production is expected in the upcoming future. To tackle this issue large efforts are being put into the development of efficient recycling technologies. The number of initiatives and projects in the field has been increasing over the years. For example, in 2022 Hydrovolt became operative, which is Europe's largest electric vehicle battery recycling plant with the ability to process 12,000 tonnes of battery packs or around 25,000 EV batteries yearly.

In addition, the New EU regulatory framework for batteries, proposed in December 2020, considers recycling as a strategic element for sustainable battery development and production (European Commission, 2020). According to the regulation's Measure 5, the recycling efficiency for Li-ion batteries targets 65% by 2025 and material recovery rates for Co, Ni, Li and Cu are defined for 2025. Measure 9 intends to set mandatory targets for the recycled content of Li, Co, Ni and Pb for new batteries put on the market in 2030 and 2035. Another important measurement (Measure 10) is establishing extended producer responsibility for batteries, which would incentivize sustainable practices along the entire life cycle of the product. These measures intend to promote the development of recycling technologies that would otherwise not advance due to the high costs.

Even though the technology for battery recycling has seen improvements in recent years, the recycling processes still do not provide a cost-effective alternative against raw material extraction (Beaudet et al., 2020) and, as some studies argue, current recycling technologies in many cases do not achieve a reduction in the life-cycle GHG emissions (Ciez and Whitacre, 2019). This is a consequence of the several issues and limitations that EV battery recycling must face.

• Impacts related to collection and transportation
• Energy-intensive processes: current state-of-the-art technologies (i.e pyrometallurgy) use high temperatures and do not yield useful results for low Cobalt chemistries like LFP or LMO (Fujita et al., 2021).
• Diversity of battery design: the lack of standardization, even for a single EV manufacturer (Gaines et al., 2018), hinders the scaling up of the recycling processes (Harper et al., 2019).
• Difficulty of disassembly: the design of battery packs is not optimized for easy disassembly (Harper et al., 2019) as adhesives and binders are used to build the EV battery (Erythropel et al., 2018).
• Low recovery rates, which hinder large-scale recycling (Gaines et al., 2018).

Overcoming the previous discussed limitations would enable to develop sustainable processes and economies of scale to boost interest in recycling. However, as mentioned previously, recycling comes only after the rest of the circular cycles are not feasible and strategies to narrow and slow loops should be prioritized, even if the ultimate step in the battery lifetime is recycling. This is especially important considering the lack of maturity of the commercial recycling processes.

Consequently, before recycling, a different alternative is encouraged by the circular economy, which is reuse. Reuse takes the form of giving the retired battery a second-life in a new application before sending the battery to the recycling facilities.

### 3.2 Second-life applications

Considering the assumed threshold of 70–80% SoH, especially with a large battery capacity, an important residual value is expected when the battery is retired from the EV. For this reason, since the last decade, second-life applications have gained attention in research and business models, as a way to generate revenue, extend the use of the battery and delay the recycling process. In fact, the interest in second-life applications rose considering the burden that EoL batteries represented for Original Equipment Manufacturers (OEM) (Olsson et al., 2018).

Besides recycling, battery reuse is the other key action promoted by European policies. The most ambitious measurement in the Battery Directive defines that retired batteries from EVs may not be considered waste if they meet the specific end-of-waste criteria, which includes an SoH check. The increased funding that second-life-related activities have received in recent years manifests this push from institutions. For example, the H2020 Advanced Light-weight BATteRy systems Optimized for fast charging, Safety, and Second-life applications (ALBATROSS) project aims to design new batteries, optimized for second-life applications.

Batteries and, in general, storage systems can be employed in a broad number of applications. The most common applications for second-life batteries are time-shifting (energy arbitrage), peak shaving, grid services, integrations of renewables, EV charge support and capacity reserve (Asian Development Bank, 2018).

Retired EV batteries can be configured in different ways for a second-life application. One of the options is to directly reuse the whole EV pack, individually or stacking several units. This strategy is more accepted by car manufacturers as their product is less manipulated, thus having less risk of failure. Stacking batteries have lower costs than other strategies that imply disassembling the pack and all internal components of the battery packs are used; thus, no waste is generated in the transition to this new life. An important issue of this strategy is related to communications since the EV BMS is not designed for second life and access to the BMS specifications is restricted by the manufacturer. In addition, the size and design of the system are not customizable, as the EV battery imposes it. Another major disadvantage of direct reuse is that the behaviour of the whole system depends on the worst module or cell that could not be changed in the transition between first and second life like in other possible configurations.

Instead of using the entire battery, single modules or even cells can be extracted and reconfigured to create a new pack optimized for the new application. This configuration offers great flexibility to design the storage system and offers better performance as the best cells or modules of the pack are selected, reducing the possibility of introducing a damage cell or module. On the other hand, the new modules with second-life cells have to be built from zero. This implies that a new module structure has to be designed, new components must be manufactured (new case, connections, sensors and BMS) and additional assembly is needed. In addition, as highlighted in Section 3.1, the design of current EV batteries makes the disassembly process complicated, relatively expensive and unsafe (Rallo et al., 2020).

Being a relatively new business model, second-life applications present several uncertainties that put into question their performance, economic profitability and environmental interest.

From the performance point of view, a source of uncertainty is their state after retirement from the vehicle. The EV market is still in the early stages and therefore, few EoL batteries can provide an understanding of the actual degradation level at their retirement. As mentioned in Section 2, second-life studies rely on a fixed threshold of 70–80% SoH for their assessment and, likely, many batteries will not have this value at EoL (Canals Casals et al., 2022). If the degradation level is higher, the technical feasibility of the second-life is reduced (Egoitz Martinez-Laserna et al., 2018b).

On the economic side, it is unclear whether these applications are as profitable as some authors have suggested due to the cost of repurposing the batteries (Haram et al., 2021). Discrepancies remain between studies that analyse the cost of battery repurposing. A recent review concluded that, depending on the estimation, the repurposing costs range between 1.29 and 55.38 €/kWh (Hossain et al., 2019). However, other studies are less optimistic. For example, a different publication showed that repurposing could cost between 87 and 360 €/kWh (Canals Casals et al., 2014).

In terms of the environmental impact, most studies conclude that the use of second-life batteries carries an associated carbon footprint reduction, compared to the use of new batteries for the same purpose. In a recent publication, a reduction in emissions of 7–31% was estimated (Kamath et al., 2020). Similarly, a 25% improvement in emissions was obtained from a Life Cycle Assessment (LCA) (Cicconi et al., 2012). Nevertheless, the environmental impact of second-life batteries is strongly linked to the expected lifetime and ageing behaviour, which is still uncertain (E. Martinez-Laserna et al., 2018a). Furthermore, these studies are based on the assumption that the second-life battery replaces a new one when in reality, this application would have not been deployed in the first place due to the high cost of the new battery (Kotak et al., 2021). Therefore, the reduction in emissions cannot be associated with the second-life battery.

In addition, the growth of EV sales also means that the same amount of batteries will be reaching EoL at some point. Considering the forecasted demand for energy storage, several sources point out that the needs of this market will be saturated with second-life batteries close to 2030–2035 (IEA, 2020; Zhao et al., 2021). Therefore, many of the retired batteries will have no place for a second life.

Besides the previous uncertainties that question the feasibility of second-life at a large scale, as represented in Fig. 3, reusing implies higher resource loops, which generally creates higher leakage and environmental impact. Therefore, the discussion on circular economy alternatives should give higher weight to the battery's first life. An alternative to second-life applications is V2G, which can extend the use of the battery while also providing similar grid services.

### 3.3 V2G

The integration of EVs into the distribution network poses several challenges for grid operators. EV charges can affect the load profile, distribution system component capacity, voltage and frequency imbalances, excessive harmonic injection, power losses and the stability of the distribution grid (Das et al., 2020). However, the growing number of EVs also opens an opportunity to develop advance power management strategies to ease these problems (Pieltain Fernandez et al., 2011). This was one of the key motivations to develop smart charging algorithms and the so-called V2G services.

Smart charging, sometimes also referred to as V1G, enables to control the charging of the battery to adapt it to the existing grid conditions. In the case of smart charging, the battery does not experience additional cycling, only the time and conditions of the charging process are modified. Smart charging can be deployed to benefit the grid by shifting the load demand, to the consumers by reducing the charge cost or to third party aggregators who trade the flexibility of the EV batteries to obtain economic benefit (Q. Wang et al., 2016b).

V2G represents a step further in the control of the EV battery and it implies additional cycling of the battery to provide grid services. Considering that EVs spend most of their time parked, around 96% of the time (Kempton and Tomic, 2005), V2G enables EV batteries to act as energy storage units, rather than a pure source of energy for the vehicle engine. In this way, the parked EV can be used to provide the same services intended for second-life businesses, such as peak shaving, energy arbitrage or frequency regulation. V2G adds a new tool for value extraction during the entire life of the EV and can help reduce the impact of the integration of the EVs on the distribution grids (Pieltain Fernandez et al., 2011), all while generating economic revenue for EV owners (Ma et al., 2012).

Even though V2G was born as a way to increase the system flexibility and generate new economic streams, it can also be positioned in the circular economy framework as a way of sharing the use of the battery between two applications: the primary automotive purpose and the secondary grid service provision. The objective of the sharing concept is to minimize the number of products required to meet the existing needs (Suárez-Eiroa et al., 2019).

There are still a few limitations to implementing V2G in real life, including the additional battery degradation, energy losses, the need for developing robust communication protocols to support V2G and the additional infrastructure needed (Noel et al., 2019). To provide V2G services, the EV needs to support and be connected to a bidirectional charger supported by a communication standard that allows a bidirectional power flow, which is more complex and represents an important investment cost (Shariff et al., 2019).

In addition, EVs cannot access the flexibility markets individually; they must do it in an aggregated form to meet the minimum required bid sizes imposed by these markets. A key stakeholder that allows this operation is the Demand Aggregator (DA). Following the nomenclature of Fig. 3, DAs are the service providers that have the tools to aggregate different assets to trade efficiently in the market. The market integration of the DAs, which is still limited in many cases due to legislative constraints (Barbero et al., 2020), is a key enabler to deploy V2G.

Nevertheless, a big challenge of V2G is often said to be the degradation caused by the additional battery use, which is highly dependent on the type and frequency of the service considered (Noel et al., 2019). Some studies suggested that V2G causes a relevant degradation of the battery, reducing its lifetime to 2–5 years depending on the service and creating the need for replacement over the lifetime of the EV (Bishop et al., 2013).

However, most works are more optimistic about the feasibility of V2G. A study on battery degradation caused by V2G concluded that extreme frequency regulation and peak load shaving (providing services every day) created an additional capacity fade of 3.62% and 5.6% respectively over 10 years. If these services were provided only occasionally, the degradation was reduced to 0.4–1.2% (D. Wang et al., 2016a). Another study developed an experimental demonstration of V2G and argued that the additional degradation caused by the services is almost negligible (Shinzaki et al., 2015).

Considering that providing driving range is the original purpose of the battery, V2G should not force an early replacement of the battery or compromise meeting the driving requirements. However, based on the premise that the EV battery can be used more intensively, this increased degradation caused by V2G (less than 6% in the studies reviewed) does not come as a limitation, but as an opportunity to deplete the value of the battery during the first life.

Until now, the circular practices reviewed are related to developing new cycles to extend the use of the battery. However, a straightforward way to increase the sustainability of EV batteries is to extend their first life as far as possible, delaying the end of the first life.

### 3.4 End of first life

An important part of the research in this area is aimed at finding the working conditions of the battery that minimize degradation and therefore extend their first life. For example, several studies propose algorithms for optimizing the charging process (Chung et al., 2020; Hoke et al., 2014).

As another alternative to extend the value of the EV battery, the option of redefining the EoL criteria for the first-life can be considered. As discussed in the introduction, currently, the functional retirement point of the battery is not estimated and the only available criterion is the 70–80% threshold. An accurate determination of the EoL can allow to extend the first life as much as possible until the battery should be retired for functional limitations and provide valuable knowledge on the state of the battery at the EoL. This action does not imply any additional investments and for that reason, this line of research has been located as the smallest circular cycle in Fig. 3.

In a recent publication, Arrinda et al. presented a methodology to calculate the EoL threshold depending on the application, for two particular use cases, a high-energy application and a high-power one (Arrinda et al., 2021). However, to the author's knowledge, no work has been developed to evaluate the driving requirements of a specific driver and estimate the functional EoL of their battery. The general framework for this estimation will be presented in this work in Section 5. To be able to estimate the functional EoL, an accurate estimation of the degradation is key. For that reason, the following section reviews existing algorithms for battery degradation estimation and prognosis.

## 4 Battery degradation estimation and prognosis

The ageing phenomenon is an unavoidable process that takes place when a battery is cycled or just stored. Degradation is a complex combination of different ageing mechanisms that create an irreversible loss of capacities in the battery (Barré et al., 2013). Among these mechanisms, the thickening of the solid electrolyte interface layer at the anode is viewed as the main cause of Li-ion battery ageing (Han et al., 2019). Other relevant degradation mechanisms in the anode include metallic lithium plating and the loss of active material (Woody et al., 2020).

Degradation is commonly divided into calendar and cycling ageing, depending on the mechanism that causes it, which can be time and the operation of the battery, respectively. While calendar ageing is mainly affected by the storage temperature and the State of Charge (SoC) (Dubarry et al., 2018b; Kassem et al., 2012; Wikner and Thiringer, 2018), in cycling ageing more variables play a role. In this case, low or high values of the average SoC, high charge and discharge currents, high Depth of Discharge (DoD) and high or low temperatures are detrimental to the battery health (Han et al., 2019; Jaguemont et al., 2016; Schmalstieg et al., 2014; Vidal et al., 2019).

One of the first challenges to estimating the degradation comes when looking at real operation conditions. The previously listed stress factors can be studied individually to understand how each one affects the degradation. However, this controlled operation is far from the real world conditions. The physical environment (temperature and humidity), where the vehicle is being used and the user's driving pattern add complexity to the degradation process (Basia et al., 2021). In addition, battery degradation is a non-linear process, with a slower degradation at the beginning of life. As the degradation increases and the battery gets closer to EoL, the battery suffers a sudden increase in ageing where the main degradation mechanism changes (this point is often referred to as the ageing knee) (Fermín-Cueto et al., 2020). Another source of complexity is the so-called path-dependency of the degradation, meaning that depending on the previous usage, even for the same stress factors, the degradation might follow a different trend (Dubarry et al., 2018a; Raj et al., 2020; Su et al., 2016). With all of this in mind, it is clear that obtaining an accurate measurement of battery degradation, especially on-board in the BMS, is a challenging task.

A common way to track the battery degradation is by estimating the SoH which is often defined as the battery capacity (Q_C_) over the nominal one (Q_nom_) as shown in Eq. (1). In some cases, often for hybrid applications where the power has a relevant impact, the SoH is alternatively defined based on the current value of the internal resistance (R_C_), considering the EoL value (R_EoL_) and the nominal one (R_nom_), as shown in Eq. (2).

(1) $$SoH=\frac{Q_{c}}{Q_{nom}}$$

(2) $$SoH=\frac{R_{EoL}-R_{c}}{R_{EoL}-R_{nom}}$$

However, these definitions individually do not give an accurate description of the state of the battery. For this reason, some authors have proposed a combination of both factors when defining the SoH (Knap et al., 2018; Lee et al., 2016). Instead of a single feature, other authors suggested using a matrix of several parameters to evaluate SoH (Yang et al., 2021).

Degradation, however, should be understood in the context of the current application, knowing that each one has its own requirements. A degradation level that might be acceptable for one case could cause another one to reach EoL. For example, a taxi driver that requires a very high range is more sensitive to a 10% capacity fade than a user who only uses the EV for short distances. However, in both cases the SoH value would be 90% (using Eq. (1)). For this reason, a new indicator called SoF has been proposed in the literature. The SoF measures the ability of the battery to serve a particular application in its current state (SoC, SoH and temperature) (Balagopal and Chow, 2015). The SoF definitions found are limited to considering the power capability of the battery and often define the SoF as a 1/0 indicator (1 when it can serve the application, 0 when it cannot) (Juang et al., 2012). Taking a 1/0 approach does not give a good picture of how far the battery is from not being functional or reaching the EoL. In this sense, an SoF definition going from 100% (at BoL) to 0% (at EoL) would allow for better management strategies. This approach is similar to the definition of the SoH given by Eq. (2), where EoL values are considered. However, the SoF should include other performance-related aspects and consider power, energy and safety-related issues.

To be able to define the SoF and predict the functional EoL, existing algorithms for SoH and RUL should be coupled with the application requirements. For that reason, the next sections review the state-of-the-art algorithms for battery state estimation and prediction.

### 4.1 State of Health estimation algorithms

The current battery capacity can only be measured in laboratories, under specific conditions and performing a full battery discharge/charge. Since this is not possible on-board, to obtain the SoH while the battery is inside the vehicle, algorithms must be developed. Current BMS estimations struggle to accurately predict the SoH of a battery, making it an open problem in research (Balasingam et al., 2020). In fact, many EV models require an external tool for estimating the SoH (e.g. Nissan's LeafSpy).

The first step for developing SoH algorithms is to find features or Health Indicators (HI) that can be measured or calculated from online data. The different HIs can be classified, based on their origin, into model-based, raw or analytically obtained ones (Basia et al., 2021). Model-based features require building a battery model, with more or less complexity. Among the different models proposed in the literature, Equivalent Circuit Models (ECM) are the most commonly employed ones for EVs (Zhang et al., 2014). The value of different ECM model parameters, such as the Open Circuit Voltage (OCV) (Fan et al., 2019), internal resistance (L. Chen et al., 2018a; Dai et al., 2009; Lee et al., 2016), diffusion resistance (Kim and Cho, 2011; Lee et al., 2016) or the diffusion time constant (Attidekou et al., 2017), have been used to build SoH algorithms. Another work proposed to use the three parameters of an ECM model, along with the value of the SoC, to evaluate the SoH (Yang et al., 2017a).

Raw values from the BMS can be directly inputted to build a SoH algorithm. A recent study considered the voltage, current and ambient temperature profiles to estimate the degradation level (Chaoui and Ibe-Ekeocha, 2017). Another algorithm was built based on the full-cycle battery discharge voltage profile (Lee and Lee, 2021). The voltage recovery, that is, the change in voltage after the EV is turned off, can be measured directly and has also been used as a HI of the battery (Baghdadi et al., 2016).

Analytically obtained HIs are derived from the data coming from the BMS. A common method to estimate the SoH is through the Incremental Capacity (IC) or Differential Voltage (DV) analysis. The voltage plateau of the discharge and charge curves turns into identifiable peaks on the IC and DV curves (Dubarry et al., 2006). Several features from these curves can be used to estimate the battery SoH, including the values and location of the different peaks (Ansean et al., 2019; Berecibar et al., 2016; Wang et al., 2017; Weng et al., 2016). Other authors have built SoH algorithms based on the Coulombic Efficiency, calculated from the discharged capacity over the charged capacity for the same cycle (Yang et al., 2018). Other possible HIs are obtained from the CC-CV (Constant Current – Constant Voltage) charge. These HIs include the initial/final voltage and currents, the charge capacity at the CC or CV periods and the CC or CV time (Z. Chen et al., 2018b; Eddahech et al., 2014; Hu et al., 2014; Zhou and Huang, 2018). Regarding discharge phases, the so-called excitation response level (Yu et al., 2020) or the changing rate of temperature (Yang et al., 2016) have been proposed as possible HIs.

When selecting HIs different factors should be considered. Firstly, the HIs should be able to correlate to the SoH accurately. A study ranked different HIs depending on how accurately they reflected the SoH based on laboratory experiments, highlighting that the best correlation was found through a HI that measured the energy discharged at constant current (Li et al., 2021). However, this situation is impossible under driving conditions. For on-board SoH estimation, the conditions for which the HIs were measured in the laboratory should be reproducible in real-world conditions. For this reason, it was suggested that HIs obtained during the CC-CV charge hold higher interest, due to the repeatability and stability of this process (Li et al., 2021; Liu and Xu, 2019). Finally, considering that laboratory degradation experiments are costly and time-consuming, the selected HIs should try to minimize the test requirements.

Once the desired HI, or combination of them is selected, a correlation with the SoH must be found. The algorithms that can be implemented in a BMS require low computational power, often taking the form of a lookup table or simple analytical expression. However, with the growth of cloud services, a new world of opportunities has arisen for battery management and analytics (Baumann et al., 2018; Li et al., 2020). Computational cost, which is one of the biggest constraints for algorithm implementation in the BMS, is now of reduced importance due to these cloud services, opening the way for more complex, Artificial Intelligence (AI) based methods to be used. Among these methods, Neural Networks (Chaoui and Ibe-Ekeocha, 2017; Yang et al., 2017a), Gaussian Process Regression (GPR) (Wang et al., 2017), Relevance Vector Machine (RVM) (Zhou and Huang, 2018) or Support Vector Regression (Z. Chen et al., 2018b; Weng et al., 2016) are found in the literature. Employing these AI-based methods, combined with an adequate selection of HIs, allows obtaining more accurate estimations of the SoH compared to traditional algorithms.

### 4.2 Remaining Useful Life (RUL) estimation

Based on the knowledge built from the SoH execution, RUL algorithms are designed to estimate the time before the batteries reach the EoL. If a robust SoH algorithm is developed, throughout the cycling of the EV battery, the degradation trends found can be correlated with the operating conditions that the battery has been subject to. In this way, the degradation can be projected into the future until reaching the EoL threshold.

Among the different algorithms for RUL estimation, Machine Learning methods have gained attention in recent years, as a way of learning complex degradation trends from on-board data (Li et al., 2019). A review of the different algorithms highlighted GPR and RVM as promising alternatives due to their nonparametric character, ability to perform probabilistic predictions and the good trade-off between accuracy and computational cost (Lucu et al., 2018). Using a probabilistic prediction, the RUL is not defined as a point but as a probability distribution of when the EoL event will take place.

However, many of the existing RUL algorithms lack essential inputs related to the future load. Some of the proposed algorithms only include the cycle number, or equivalently, the charge throughput (Chen et al., 2022; Liu and Chen, 2019). Other works include features like the temperature or the DoD. For example, a cycling ageing prediction is proposed using the temperature, charge and discharge c-rates, the average SoC and the DoD (Lucu et al., 2020). Nevertheless, this type of forecast does not consider the variable operating conditions of the battery during its lifetime. As highlighted by a recent review, the variability of operational conditions should be captured through the use of distributional information (von Bülow and Meisen, 2023). Up to date, only a few works have proposed the use of inputs contained in histograms to forecast the SoH. One of these works trained a GPR model with, along with other inputs, the time elapsed during which certain conditions are met (specific temperature, voltage and current ranges) (Richardson et al., 2019). Another recent study used histograms to extract relevant and independent features and to train different Machine Learning models (Zhang et al., 2022). A different approach is presented in a study that obtains some of the inputs from applying the Rainflow Counting algorithm, which is commonly used in fatigue analysis (Nuhic et al., 2018).

Although the latter histogram-based studies allow capturing, to some extent, the complexity of the operational conditions, all the existing RUL algorithms have something in common: they consider the EoL threshold of 70–80% SoH, which oversimplifies the estimation. To overcome this limitation, in the following section the authors propose a framework to estimate the functional EoL and, therefore, a more realistic RUL.

## 5 Results

As a result of the performed review, the authors have highlighted an important gap in the literature: the functional EoL point of the battery is not estimated based on the use of the battery. The result of this study is precisely to provide the methodological framework to fill this gap. The authors, henceforth, highlight the issues that should be further investigated and propose a methodology to define the battery EoL based on functional requirements. The opportunities for using the proposed methodology are discussed at the end of this section.

**Fig. 4:**
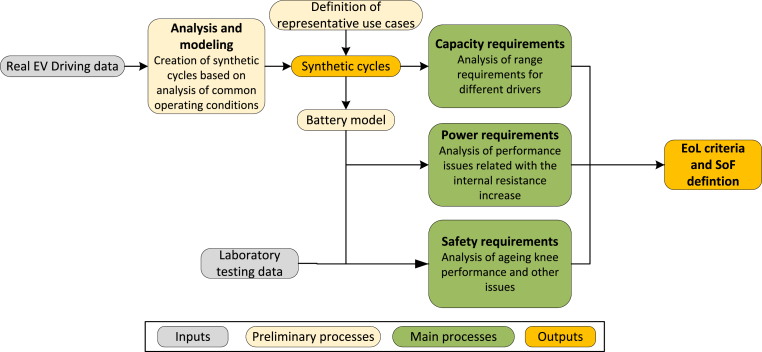
Methodological framework for the driving requirements.

The first step is to analyse the main limitations that could force a battery to reach the EoL for different use cases. This line of research needs to be supported by evidence from an extensive analysis of the first-life requirements and battery performance under different conditions to determine whether the commonly assumed threshold could be redefined for each user. Fig. 4 (Analysis and modelling), presents the different real driving patterns of users that must be analysed for this assessment. Considering that access to BMS data is often restricted due to confidentiality issues, the use of synthetic cycles can be considered. These cycles, in combination with battery modelling and additional laboratory data, can be used to understand the functional limitations of the batteries during driving. If access to a large dataset of driving data is accessible, the steps marked as preliminary in the figure can be omitted. In particular, the following aspects must be further addressed, which have been represented in green in Fig. 4.

• **Capacity requirements**: different driving patterns, representative of the population, should be analysed to evaluate the feasibility of reducing the EoL SoH threshold. Depending on the driving style, EV model and environmental the specific energy consumption changes. First of all, the road type and driving style should be considered. High speeds common in highway driving (Wager et al., 2016) and stop-start conditions of urban driving can increase the specific energy consumption (Al-Wreikat et al., 2021). In addition, the need for auxiliary vehicle services changes depending on the ambient temperature. In this sense, in extreme temperature environments, the specific energy consumption increases significantly (Al-Wreikat et al., 2022). Finally, the efficiency of the traction system should also be considered. Aspects like the weight (largely affected by the battery capacity), the EV design that affects the aerodynamic or the on-board power electronics efficiency influence the efficiency of the system. Depending on these aspects, the minimum capacity requirements until the EoL can be obtained.
• **Power requirements**: knowing that the internal resistance increase reduces the available power, a highly degraded battery could not be able to cover the required driving needs. Higher currents can force to reach the operating limits of the battery faster if the current peaks take place at low operating voltages. In addition, under cold temperatures the internal resistance increases even more and therefore, until the battery does not self-heat up, the power limitation may be a key factor to evaluate. An additional consequence of the internal resistance increase is the increased heat generation, following the Joules effect. The power of the cooling system of the EV, if it has one, directly defines the amount of heat that can be evacuated to maintain the battery in a safe operating area. If the heat generation is excessive additional power limitations may be imposed by the BMS. Therefore, the temperature, current and common DoD of the driving trips, along with the characteristics of the cooling system, should be understood to translate the power requirements into the internal resistance EoL value.
• **Safety requirements**: Besides the previously discussed power limitation related to the increased heat generation, other safety issues should be considered. As the internal resistance increases, a lack of effective cooling may cause high operating temperatures, which can lead to the exothermic decomposition of electrodes and electrolyte materials and separator shrinkage that can induce an internal short circuit (Zhang et al., 2018). Several events caused by degradation mechanisms (e.g. dendrite growth or lithium loss side reactions) and unbalances can lead to EV fire (Hu et al., 2021). These aspects are especially relevant once the battery reaches a critical point in degradation: the ageing knee. Studies have confirmed that the battery eventually suffers a change in the dominant ageing process, which drastically increases the degradation rate. At this point, safety-related issues, such as short circuits, are more common (Braco et al., 2020). Several experimental studies argue that the ageing knee occurs before 80% SoH (Egoitz Martinez-Laserna et al., 2018b) and others show that, depending on the usage and chemistry, the ageing knee occurs after 80% (Attia et al., 2022). Nevertheless, most studies use non-automotive cells which show different ageing characteristics than automotive ones (Braco et al., 2020). This last reference tested actual EV modules to evaluate the nonlinear ageing behaviour and found that it took place well below 80% SoH, even at 40–50% in some cases. Therefore, safety-related aspects should be studied as the battery ages to evaluate when they can force the battery EoL.

Evaluating the previous points allows assessing whether the fixed EoL criterion is indeed restrictive and whether the battery EoL SoH could fall below 70–80% while still being functional and safe. It also can serve to define the issues that the SoF should include so that it accurately represents the functionality of the battery.

**Fig. 5:**
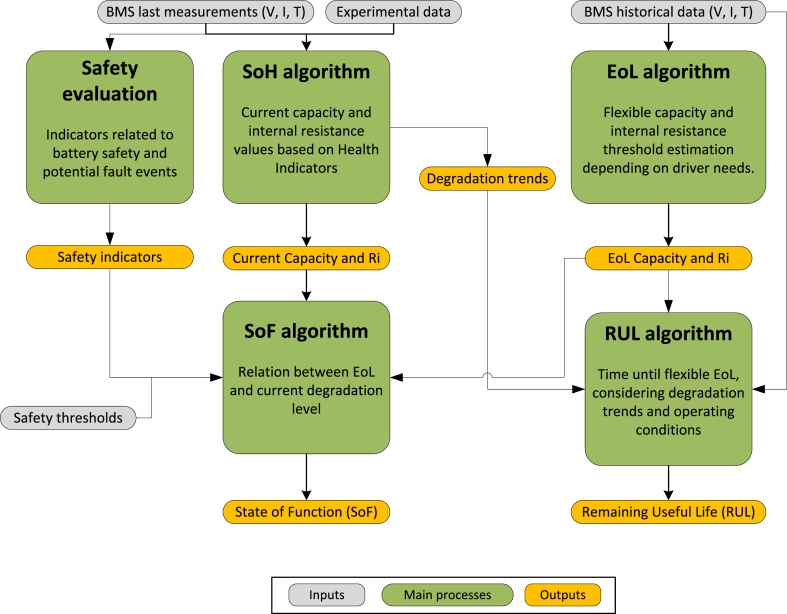
Methodological framework for the functional EoL definition.

Unlike having a universal fixed threshold for all battery sizes and driving requirements, the optimum EoL should be individually defined based on the functional limitations of each driver and the characteristics of the vehicle. An individual analysis should be made to evaluate, on one hand, the requirements that the driver expects from the battery until its EoL and, on the other hand, the degradation caused by their driving pattern and environmental conditions. With this in mind, the authors propose a methodology to determine the functional EoL for each case, based on information that is logged on the BMS. The EoL definition framework, represented in Fig. 5, is composed of five steps.

1. **EoL requirements:** the first step is to analyse the functionalities that the driver requests from the battery. The historical data from the BMS can be used to estimate the driving range and power that the battery should provide until its EoL. The power needs to be translated into the threshold internal resistance value that cannot be surpassed, considering that the battery should work on the defined voltage and temperature operation range. Therefore, the output of this step is the functional EoL threshold in terms of capacity and internal resistance.
2. **SoH estimation:** HIs extracted from the BMS measurements should be correlated to the current capacity and internal resistance, based on degradation models obtained from experimental data. The output of this step is the current capacity and internal resistance.
3. **Safety evaluation:** in the BMS, safety-related indicators should be obtained to evaluate potential faults in the battery. These indicators can be obtained whenever the EV starts operating, before corrective actions, such as cooling, are put into place. It is out of the scope of this study to define the indicators that should be obtained. However, the reader can refer to the literature for further information, for example, up to 14 indicators to evaluate the safety of the battery, including lithium dendrite resistance or separator ageing have been proposed for this purpose (Yu Wen et al., 2015). However, other safe operation indicators can be directly obtained from the BMS cell/pack voltage, temperature, current, and SoC (Rezvanizaniani et al., 2014). The outputs of this step are the safety indicators.
4. **SoF estimation:** the SoF provides an indicator for how far the battery is from the functional EoL threshold. Therefore SoF will relate the current state of the battery to the defined EoL threshold values. At BoL, the SoF has a value of 1, which decreases as the battery degrades. The SoF will be set to 0 whenever one of the constraints is met (capacity, internal resistance or safety), meaning that the battery has reached EoL. A possible definition for the SoF is given below (Eqs. (3), (4), (5), (6))). Eqs. (4), (5)) relate the current health of the battery (step 2) to the defined threshold values (step 1). Eq. (6) considers safety-related aspects by comparing the current value of the safety indicators ($X_{i}$) to the threshold values, which are inputs to the analysis.
  (3) $$SoF=\min\left(SoF_{Capacity},SoF_{Ri},SoF_{Safety}\right)$$
  (4) $$SoF_{Capacity}=\frac{C-C_{EoL}}{{C_{BoL}-C}_{EoL}}$$
  (5) $$SoF_{Ri}=\frac{Ri_{EoL}-Ri}{Ri_{EoL}-Ri_{BoL}}$$
  (6) $$SoF_{safety}=\min_{i=1\ldots n}\left(\frac{X_{i}-X_{i,EoL}}{X_{i.BoL}-X_{i,EoL}}\right)$$

5. **RUL estimation:** the trends found through the SoH algorithm allow to project the degradation until it reaches the flexible EoL defined in step 1 (the SoF reaches 0), which represents the functional RUL.

**Fig. 6:**
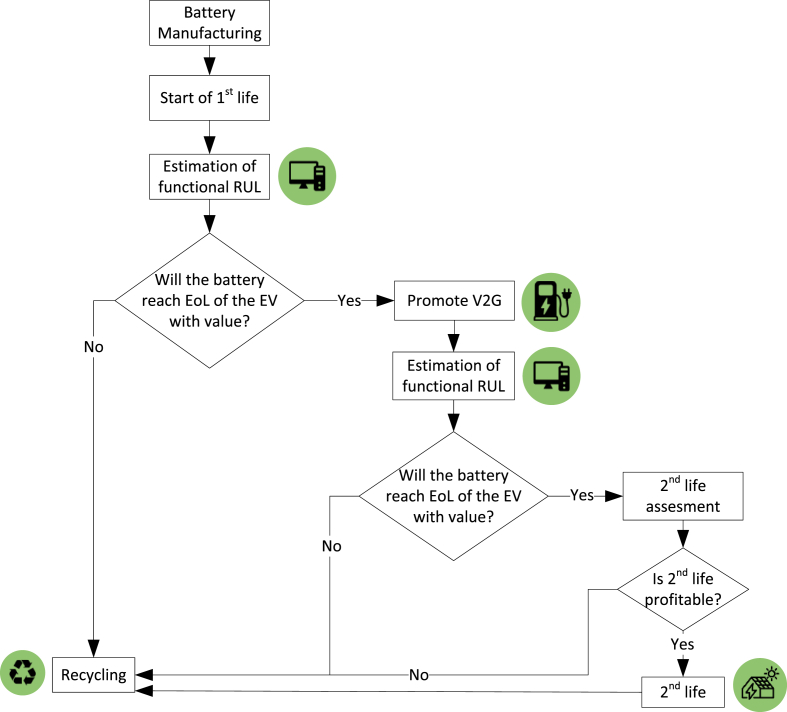
Battery use decision diagram.

Implementing the previous methodology as a software service on-board can be a useful tool for EV drivers and other relevant stakeholders. It can be useful to make a preliminary study on the technical and economical feasibility of different second-life alternatives, while the battery is still inside the EV. Tracking the SoF can also give insight into the intensity of the V2G services that can be provided to avoid an early retirement or the underuse of the battery and to estimate the requirement for battery replacement. Fig. 6 shows how the functional EoL estimation could be used to maximize the usage of a particular battery.

After the BMS has received sufficient data to analyse the usage pattern of the EV driver, a first estimation of the functional RUL can be performed. This prediction can be contrasted with the expected lifetime of the vehicle. If a residual value in the battery is anticipated, V2G, to a lower or higher degree, can be promoted to intensify the battery usage.

If V2G is performed, a second estimation of the functional RUL can be carried out to analyse whether the battery will still hold value at the EoL under this new working pattern. In this case, the residual value after the first life can be extracted through a second-life application, as long as it is technically and economically feasible.

This preliminary assessment for the second-life could be performed while the battery is still in the EV. The possibility of performing the assessment on board will depend on the confidentiality of the data that car manufacturers may not be willing to share. However, if this is not the case, or if the own car manufacturer is the one responsible for the second-life application, this early assessment can provide several benefits too. On the one hand, obtaining early information, before the EV EoL, could be used for planning purposes. And, on the other hand, the historical usage pattern and estimated SoH at EoL could be used to reduce the need for testing the EoL battery, which is a costly and time-consuming process (Hossain et al., 2019).

## 6 Discussion

This work has reviewed different processes that can be encompassed in the circular economy framework. Some of these processes are extensively presented in the literature as an opportunity for environmental impact reduction (reuse and recycling). Other processes, although technically understood, are often omitted in this assessment (V2G and capacity reduction). Finally, the result of the review has been the exploration of a new process necessary for sustainable decision-making (functional EoL definition).

Understanding which process should be prioritized requires a deeper and case-specific analysis. However, some generalizations can be made considering key criteria.

• Criteria 1: reduction of the pressure on raw material extraction
• Criteria 2: reduction of the costs for the battery owner
• Criteria 3: provision of additional value (i.e. grid services)

Table 1 shows the circular processes considered along with the criteria proposed indicating whether they produce a positive effect (+), a negative one (−) or no effect (blank), compared with the baseline scenario where the battery is manufactured, used to 70–80% SoH in the EV and then disposed of.

**Table 1:** Comparison of circular processes.

	1. Costs	2. Raw materials	3. Additional value
Reduction of the battery capacity	+	+	
Functional EoL definition	+	+	
V2G	+		+
Second life			+
Recycling	–	+	

Strategies that favour the first stages of the battery lifecycle seem more compelling. Choosing to reduce the battery's nominal capacity to lower values, reduces the cost and the use of raw materials for manufacturing the battery. However, it has already been discussed that market trends are difficult to shift, which has motivated the analysis of the other circular processes.

The proposed process of defining a functional EoL helps to avoid an early retirement of the battery, which delays the manufacturing of a new battery for the same driver. In this way, throughout its lifetime the demand for batteries from the same driver would be reduced, generating a positive impact on the cost and raw material extraction.

Both V2G and the second life allow providing grid services and generate no impact on the raw material extraction, with the assumption that the services provided do not require an additional product in the baseline scenario. However, V2G can be more cost beneficial than the second-life, considering that, as discussed previously, V2G only requires the purchase of a bidirectional charger.

Recycling appears to be the least interesting process according to the criteria defined. Nevertheless, although recycling currently does not hold an economic interest, it is a necessary action to guarantee a circular economy and therefore, a key element for investment and research efforts.

Therefore, this review suggests putting further effort into the vehicle's first life. This idea, although well aligned with the circular economy guidelines presented at the beginning of Section 2, differs from other circular economy reviews. As highlighted in the introduction, existing reviews for EV batteries remain mainly focused on the last stages of the battery lifetime (reuse and recycling).

The same tendency to promote recycling and second-life applications is also found in European policies (i.e. Battery Directive). Although investment and policy efforts should still consider these processes, following the idea of this study, efforts should be put into defining a stronger case for V2G. This includes defining safe communication protocols, legislation around demand aggregators, improvements of the electricity grid, technical developments of control algorithms, business models and a communication network between all actors (Guille and Gross, 2009; Uddin et al., 2018).

## 7 Conclusions

The increase in EV sales forces to adopt sustainable practices to reduce the environmental impact of their Li-ion batteries, especially considering the current trend of increased battery capacity. This work highlighted an important environmental problem related to the underuse of the battery and analysed the existing and promising sources of circularity that could best overcome it.

The study has shown how current EV batteries are, in most cases, far larger than the current driving requirements. In addition, the EoL of these batteries is inadequately determined and assumed to be at 70–80% SoH for all cases, regardless of the user requirements or their nominal capacity. These two factors are translated into the underuse of the battery and lack of correct information for decision-making that hinder the sustainability of EVs.

The literature often considers battery reuse and recycling as the key elements to promote sustainability. However, this review highlights, in accordance with circular economy guidelines, that further effort should be put into the first stages of the battery lifetime. The first solution to avoid underuse would be to reduce the nominal capacity of the batteries, which are in most cases oversized considering usual range needs. Alternatively, V2G provides a valuable tool to increase the usage of the battery and provide grid services while delivering higher economic profit than second-life batteries. Finally, recycling, although currently not economically profitable, is the unavoidable last step of the lifetime to guarantee a circular economy.

The possibility of extending the battery's first life until a functional point has been highlighted as an under looked alternative that holds a high potential to improve the sustainability of EVs. To do so, the 70–80% SoH EoL criteria currently applied should be revised and a functional threshold should be defined for each EV, making sure that the battery is used for as long as possible in the EV while ensuring that it meets functional and safety aspects. In this sense, the result of the study is a framework that aims to estimate a functional EoL. To do so, the use of the SoF is proposed, which provides a better indicator of the usability of the battery than the SoH, by relating the current state of the battery to the functional requirements of each driver.

Further work is needed to confirm in which cases the battery capacity can be reduced and the EoL postponed below 70–80% SoH. This study presents future lines of research, which include the evaluation of the capacity needs of several drivers under different environmental conditions, the study of the increase in internal resistance that limits the battery power and safety-related aspects. Nevertheless, previous studies are optimistic regarding the functionality of EV batteries below the fixed threshold and, if confirmed, this line of research can provide an important tool for appropriate decision-making to extract all the value of the battery during the first-life, avoid underuse and reduce their environmental impact. Future work should include the evaluation of the EoL requirements and the application of the proposed framework to different EV users.

Considering the results of the review, research, investment and policy efforts supporting the maximization of the battery's first life should be prioritized. In this sense, reducing battery capacities should be pursued, V2G should be encouraged over promoting battery reuse and further effort should be put into extending the battery's first life as much as possible. These actions are key to guarante that the electrification of the transportation system does not pose a larger environmental problem than what it aims to solve.

## Funding

This project has received funding from the European Union’s 10.13039/100010661Horizon 2020 research and innovation program under grant agreement No. 963580. This funding includes funds to support research work and open-access publications.

## Declaration of competing interest

The authors declare that they have no known competing financial interests or personal relationships that could have appeared to influence the work reported in this paper.

## Data Availability

No data was used for the research described in the article.

## References

[bib1] Acar C., Dincer I. (2020). The potential role of hydrogen as a sustainable transportation fuel to combat global warming. Int. J. Hydrogen Energy.

[bib2] Al-Wreikat Y., Serrano C., Sodré J.R. (2021). Driving behaviour and trip condition effects on the energy consumption of an electric vehicle under real-world driving. Appl. Energy.

[bib3] Al-Wreikat Y., Serrano C., Sodré J.R. (2022). Effects of ambient temperature and trip characteristics on the energy consumption of an electric vehicle. Energy.

[bib4] Ali H., Khan H.A., Pecht M.G. (2021). Circular economy of Li batteries: technologies and trends. J. Energy Storage.

[bib5] Ansean D., Garcia V.M., Gonzalez M., Blanco-Viejo C., Viera J.C., Pulido Y.F., Sanchez L. (2019). Lithium-ion battery degradation indicators via incremental capacity analysis. IEEE Trans. Ind. Appl..

[bib6] Arrinda M., Oyarbide M., Macicior H., Muxika E., Popp H., Jahn M., Ganev B., Cendoya I. (2021). Application dependent end-of-life threshold definition methodology for batteries in electric vehicles. Batteries.

[bib7] Asian Development Bank (2018).

[bib8] Attia P.M., Bills A., Brosa Planella F., Dechent P., dos Reis G., Dubarry M., Gasper P., Gilchrist R., Greenbank S., Howey D., Liu O., Khoo E., Preger Y., Soni A., Sripad S., Stefanopoulou A.G., Sulzer V. (2022). Review—“Knees” in lithium-ion battery aging trajectories. J. Electrochem. Soc..

[bib9] Attidekou P.S., Wang C., Armstrong M., Lambert S.M., Christensen P.A. (2017). A new time constant approach to online capacity monitoring and lifetime prediction of lithium ion batteries for electric vehicles (EV). J. Electrochem. Soc..

[bib10] Baars J., Domenech T., Bleischwitz R., Melin H.E., Heidrich O. (2021). Circular economy strategies for electric vehicle batteries reduce reliance on raw materials. Nat. Sustain..

[bib11] Baghdadi I., Briat O., Gyan P., Vinassa J.M. (2016). State of health assessment for lithium batteries based on voltage–time relaxation measure. Electrochim. Acta.

[bib12] Balagopal B., Chow M.-Y. (2015). 2015 IEEE 13th International Conference on Industrial Informatics (INDIN).

[bib13] Balasingam B., Ahmed M., Pattipati K. (2020). Battery management systems—challenges and some solutions. Energies.

[bib14] Barbero M., Corchero C., Canals Casals L., Igualada L., Heredia F.-J. (2020). Critical evaluation of European balancing markets to enable the participation of Demand Aggregators. Appl. Energy.

[bib15] Barré A., Deguilhem B., Grolleau S., Gérard M., Suard F., Riu D. (2013). A review on lithium-ion battery ageing mechanisms and estimations for automotive applications. J. Power Sources.

[bib16] Basia A., Simeu-Abazi Z., Gascard E., Zwolinski P. (2021). Review on State of Health estimation methodologies for lithium-ion batteries in the context of circular economy. CIRP J. Manufact. Sci. Tech..

[bib17] Bauer C., Hofer J., Althaus H.-J., Del Duce A., Simons A. (2015). The environmental performance of current and future passenger vehicles: life cycle assessment based on a novel scenario analysis framework. Appl. Energy.

[bib18] Baumann M., Rohr S., Lienkamp M. (2018). Thirteenth International Conference on Ecological Vehicles and Renewable Energies (EVER).

[bib19] Baure G., Dubarry M. (2019). Synthetic vs. Real driving cycles: a comparison of electric vehicle battery degradation. Batteries.

[bib20] Beaudet A., Larouche F., Amouzegar K., Bouchard P., Zaghib K. (2020). Key challenges and opportunities for recycling electric vehicle battery materials. Sustainability.

[bib21] Berecibar M., Devriendt F., Dubarry M., Villarreal I., Omar N., Verbeke W., Van Mierlo J. (2016). Online state of health estimation on NMC cells based on predictive analytics. J. Power Sources.

[bib22] Bicer Y., Dincer I. (2018). Life cycle environmental impact assessments and comparisons of alternative fuels for clean vehicles. Resour. Conserv. Recycl..

[bib23] Bishop J.D.K., Axon C.J., Bonilla D., Tran M., Banister D., McCulloch M.D. (2013). Evaluating the impact of V2G services on the degradation of batteries in PHEV and EV. Appl. Energy.

[bib24] Bobba S., Mathieux F., Blengini G.A. (2019). How will second-use of batteries affect stocks and flows in the EU? A model for traction Li-ion batteries. Resour. Conserv. Recycl..

[bib25] Bocken N.M.P., de Pauw I., Bakker C., van der Grinten B. (2016). Product design and business model strategies for a circular economy. J. Indus. Prod. Eng..

[bib26] Bonges H.A., Lusk A.C. (2016). Addressing electric vehicle (EV) sales and range anxiety through parking layout, policy and regulation. Transport. Res. Pol. Pract..

[bib27] Braco E., San Martín I., Berrueta A., Sanchis P., Ursúa A. (2020). Experimental assessment of cycling ageing of lithium-ion second-life batteries from electric vehicles. J. Energy Storage.

[bib28] Canals Casals L., Amante García B., González Benítez M.M., Ayuso Muñoz J.L., Yagüe Blanco J.L., Capuz-Rizo S.F. (2014). Lecture Notes in Management and Industrial Engineering, Lecture Notes in Management and Industrial Engineering.

[bib29] Canals Casals L., Rodríguez M., Corchero C., Carrillo R.E. (2019). Evaluation of the end-of-life of electric vehicle batteries according to the state-of-health. WE!.

[bib30] Canals Casals L., Etxandi-Santolaya M., Bibiloni-Mulet P.A., Corchero C., Trilla L. (2022).

[bib31] Catelani M., Ciani L., Fantacci R., Patrizi G., Picano B. (2021). Remaining useful life estimation for prognostics of lithium-ion batteries based on recurrent neural network. IEEE Trans. Instrum. Meas..

[bib32] Chaoui H., Ibe-Ekeocha C.C. (2017). State of charge and state of health estimation for lithium batteries using recurrent neural networks. IEEE Trans. Veh. Technol..

[bib33] Chen X., Shen W., Vo T.T., Cao Z., Kapoor A. (2012). 2012 10th International Power & Energy Conference (IPEC).

[bib34] Chen L., Lü Z., Lin W., Li J., Pan H. (2018). A new state-of-health estimation method for lithium-ion batteries through the intrinsic relationship between ohmic internal resistance and capacity. Measurement.

[bib35] Chen Z., Sun M., Shu X., Xiao R., Shen J. (2018). Online state of health estimation for lithium-ion batteries based on support vector machine. Appl. Sci..

[bib36] Chen D., Meng J., Huang H., Wu J., Liu P., Lu J., Liu T. (2022). An empirical-data hybrid driven approach for remaining useful life prediction of lithium-ion batteries considering capacity diving. Energy.

[bib37] Chung C.-H., Jangra S., Lai Q., Lin X. (2020). Optimization of electric vehicle charging for battery maintenance and degradation management. IEEE Trans. Transp. Electrific..

[bib38] Cicconi P., Landi D., Morbidoni A., Germani M. (2012). 2012 IEEE International Energy Conference and Exhibition (ENERGYCON).

[bib39] Ciez R.E., Whitacre J.F. (2019). Examining different recycling processes for lithium-ion batteries. Nat. Sustain..

[bib40] Cremades L., Canals Casals L. (2022). Analysis of the future of mobility: the battery electric vehicle seems just a transitory alternative. Energies.

[bib41] Dai Haifeng, Xuezhe Wei, Sun Zechang (2009). 2009 IEEE Vehicle Power and Propulsion Conference.

[bib42] Dalla Chiara B., Deflorio F., Pellicelli M., Castello L., Eid M. (2019). Perspectives on electrification for the automotive sector: a critical review of average daily distances by light-duty vehicles, required range, and economic outcomes. Sustainability.

[bib43] Das H.S., Rahman M.M., Li S., Tan C.W. (2020). Electric vehicles standards, charging infrastructure, and impact on grid integration: a technological review. Renew. Sustain. Energy Rev..

[bib44] Dubarry M., Svoboda V., Hwu R., Yann Liaw B. (2006). Incremental capacity analysis and close-to-equilibrium OCV measurements to quantify capacity fade in commercial rechargeable lithium batteries. Electrochem. Solid State Lett..

[bib45] Dubarry M., Baure G., Devie A. (2018). Durability and reliability of EV batteries under electric utility grid operations: path dependence of battery degradation. J. Electrochem. Soc..

[bib46] Dubarry M., Qin N., Brooker P. (2018). Calendar aging of commercial Li-ion cells of different chemistries – a review. Current Opinion in Electrochemistry.

[bib47] Eddahech A., Briat O., Vinassa J.-M. (2014). Determination of lithium-ion battery state-of-health based on constant-voltage charge phase. J. Power Sources.

[bib48] Ellen MacArthur Foundation (2013). Towards a circular economy. J. Ind. Ecol..

[bib49] Erythropel H.C., Zimmerman J.B., de Winter T.M., Petitjean L., Melnikov F., Lam C.H., Lounsbury A.W., Mellor K.E., Janković N.Z., Tu Q., Pincus L.N., Falinski M.M., Shi W., Coish P., Plata D.L., Anastas P.T. (2018). The Green ChemisTREE: 20 years after taking root with the 12 principles. Green Chem..

[bib50] European Commission (2015).

[bib51] European Commission (2020).

[bib52] Fan J., Zou Y., Zhang X., Guo H. (2019). A novel State of Health estimation method for Lithium-ion battery in electric vehicles. J. Phys.: Conf. Ser..

[bib53] Fermín-Cueto P., McTurk E., Allerhand M., Medina-Lopez E., Anjos M.F., Sylvester J., dos Reis G. (2020). Identification and machine learning prediction of knee-point and knee-onset in capacity degradation curves of lithium-ion cells. Energy and AI.

[bib54] Fujita T., Chen H., Wang K., He C., Wang Y., Dodbiba G., Wei Y. (2021). Reduction, reuse and recycle of spent Li-ion batteries for automobiles: a review. Int. J. Miner. Metall. Mater..

[bib55] Gaines L., Richa K., Spangenberger J. (2018). Key issues for Li-ion battery recycling. MRS Energy & Sustainability.

[bib56] Guille C., Gross G. (2009). A conceptual framework for the vehicle-to-grid (V2G) implementation. Energy Pol..

[bib57] Han X., Lu L., Zheng Y., Feng X., Li Z., Li J., Ouyang M. (2019). A review on the key issues of the lithium ion battery degradation among the whole life cycle. eTransportation.

[bib58] Haram M.H.S.M., Lee J.W., Ramasamy G., Ngu E.E., Thiagarajah S.P., Lee Y.H. (2021). Feasibility of utilising second life EV batteries: applications, lifespan, economics, environmental impact, assessment, and challenges. Alex. Eng. J..

[bib59] Harper G., Sommerville R., Kendrick E., Driscoll L., Slater P., Stolkin R., Walton A., Christensen P., Heidrich O., Lambert S., Abbott A., Ryder K., Gaines L., Anderson P. (2019). Recycling lithium-ion batteries from electric vehicles. Nature.

[bib60] Hoke A., Brissette A., Smith K., Pratt A., Maksimovic D. (2014). Accounting for lithium-ion battery degradation in electric vehicle charging optimization. IEEE J. Emerg. Sel. Topics Power Electron..

[bib61] Hossain E., Murtaugh D., Mody J., Faruque H.M.R., Haque Sunny MdS., Mohammad N. (2019). A comprehensive review on second-life batteries: current state, manufacturing considerations, applications, impacts, barriers & potential solutions, business strategies, and policies. IEEE Access.

[bib62] Hu C., Jain G., Zhang P., Schmidt C., Gomadam P., Gorka T. (2014). Data-driven method based on particle swarm optimization and k-nearest neighbor regression for estimating capacity of lithium-ion battery. Appl. Energy.

[bib63] Hu G., Huang P., Bai Z., Wang Q., Qi K. (2021). Comprehensively analysis the failure evolution and safety evaluation of automotive lithium ion battery. eTransportation.

[bib64] IEA (2020).

[bib65] International Energy Agency (IEA) (2022).

[bib66] Jaguemont J., Boulon L., Dubé Y. (2016). A comprehensive review of lithium-ion batteries used in hybrid and electric vehicles at cold temperatures. Appl. Energy.

[bib67] Janota L., Králík T., Knápek J. (2020). Second life batteries used in energy storage for frequency containment reserve service. Energies.

[bib68] Juang L.W., Kollmeyer P.J., Jahns T.M., Lorenz R.D. (2012). 2012 IEEE Energy Conversion Congress and Exposition (ECCE).

[bib69] Kamath D., Shukla S., Arsenault R., Kim H.C., Anctil A. (2020). Evaluating the cost and carbon footprint of second-life electric vehicle batteries in residential and utility-level applications. Waste Manag..

[bib70] Kassem M., Bernard J., Revel R., Pélissier S., Duclaud F., Delacourt C. (2012). Calendar aging of a graphite/LiFePO4 cell. J. Power Sources.

[bib71] Kawamoto R., Mochizuki H., Moriguchi Y., Nakano T., Motohashi M., Sakai Y., Inaba A. (2019). Estimation of CO2 emissions of internal combustion engine vehicle and battery electric vehicle using LCA. Sustainability.

[bib72] Kempton W., Tomic J. (2005). Vehicle-to-grid power fundamentals: calculating capacity and net revenue. J. Power Sources.

[bib73] Kim J., Cho B.H. (2011). State-of-Charge estimation and state-of-health prediction of a Li-ion degraded battery based on an EKF combined with a per-unit system. IEEE Trans. Veh. Technol..

[bib74] Knap V., Auger D., Propp K., Fotouhi A., Stroe D.-I. (2018). Concurrent real-time estimation of state of health and maximum available power in lithium-sulfur batteries. Energies.

[bib75] Kotak Y., Marchante Fernández C., Canals Casals L., Kotak B.S., Koch D., Geisbauer C., Trilla L., Gómez-Núñez A., Schweiger H.-G. (2021). End of electric vehicle batteries: reuse vs. Recycle. Energies.

[bib76] Kurdve M., Zackrisson M., Johansson M.I., Ebin B., Harlin U. (2019). Considerations when modelling EV battery circularity systems. Batteries.

[bib77] Laurikko J., Granström R., Haakana A. (2013).

[bib78] Lee J.-H., Lee I.-S. (2021). Lithium battery SOH monitoring and an SOC estimation algorithm based on the SOH result. Energies.

[bib79] Lee H., Park J., Kim J. (2016).

[bib80] Li Y., Liu K., Foley A.M., Zülke A., Berecibar M., Nanini-Maury E., Van Mierlo J., Hoster H.E. (2019). Data-driven health estimation and lifetime prediction of lithium-ion batteries: a review. Renew. Sustain. Energy Rev..

[bib81] Li W., Rentemeister M., Badeda J., Jöst D., Schulte D., Sauer D.U. (2020). Digital twin for battery systems: cloud battery management system with online state-of-charge and state-of-health estimation. J. Energy Storage.

[bib82] Li Y., Stroe D.-I., Cheng Y., Sheng H., Sui X., Teodorescu R. (2021). On the feature selection for battery state of health estimation based on charging–discharging profiles. J. Energy Storage.

[bib83] Lieder M., Rashid A. (2016). Towards circular economy implementation: a comprehensive review in context of manufacturing industry. J. Clean. Prod..

[bib84] Liu J., Chen Z. (2019). Remaining useful life prediction of lithium-ion batteries based on health indicator and Gaussian process regression model. IEEE Access.

[bib85] Liu W., Xu Y. (2019). 2019 IEEE 3rd Conference on Energy Internet and Energy System Integration (EI2).

[bib86] Lowell D., Huntington A. (2021).

[bib87] Lu R., Yang A., Xue Y., Xu L., Zhu C. (2010). Analysis of the key factors affecting the energy efficiency of batteries in electric vehicle. World Electric Vehicle Journal.

[bib88] Lucu M., Martinez-Laserna E., Gandiaga I., Camblong H. (2018). A critical review on self-adaptive Li-ion battery ageing models. J. Power Sources.

[bib89] Lucu M., Martinez-Laserna E., Gandiaga I., Liu K., Camblong H., Widanage W.D., Marco J. (2020). Data-driven nonparametric Li-ion battery ageing model aiming at learning from real operation data - Part B: cycling operation. J. Energy Storage.

[bib90] Ma Y., Houghton T., Cruden A., Infield D. (2012). Modeling the benefits of vehicle-to-grid technology to a power system. IEEE Trans. Power Syst..

[bib91] Martinez-Laserna E., Gandiaga I., Sarasketa-Zabala E., Badeda J., Stroe D.-I., Swierczynski M., Goikoetxea A. (2018). Battery second life: hype, hope or reality? A critical review of the state of the art. Renew. Sustain. Energy Rev..

[bib92] Martinez-Laserna Egoitz, Sarasketa-Zabala E., Villarreal Sarria I., Stroe D.-I., Swierczynski M., Warnecke A., Timmermans J.-M., Goutam S., Omar N., Rodriguez P. (2018). Technical viability of battery second life: a study from the ageing perspective. IEEE Trans. Ind. Appl..

[bib93] Melliger M.A., van Vliet O.P.R., Liimatainen H. (2018). Anxiety vs reality – sufficiency of battery electric vehicle range in Switzerland and Finland. Transport. Res. Transport Environ..

[bib94] Mossali E. (2020). Lithium-ion batteries towards circular economy: a literature review of opportunities and issues of recycling treatments. J. Environ. Manag..

[bib95] Noel L., Zarazua de Rubens G., Kester J., Sovacool B.K. (2019). Vehicle-to-Grid.

[bib96] Noel L., Zarazua de Rubens G., Kester J., Sovacool B.K. (2020). Understanding the socio-technical nexus of Nordic electric vehicle (EV) barriers: a qualitative discussion of range, price, charging and knowledge. Energy Pol..

[bib97] Nuhic A., Bergdolt J., Spier B., Buchholz M., Dietmayer K. (2018). Battery health monitoring and degradation prognosis in fleet management systems. WE!.

[bib98] Olsson L., Fallahi S., Schnurr M., Diener D., van Loon P. (2018). Circular business models for extended EV battery life. Batteries.

[bib99] Pagliaro M., Meneguzzo F. (2019). Lithium battery reusing and recycling: a circular economy insight. Heliyon.

[bib101] Pieltain Fernandez L., Gomez San Roman T., Cossent R., Mateo Domingo C., Frias P. (2011). Assessment of the impact of plug-in electric vehicles on distribution networks. IEEE Trans. Power Syst..

[bib102] Plötz P., Sprei F. (2021). Variability of daily car usage and the frequency of long-distance driving. Transport. Res. Transport Environ..

[bib103] Plötz P., Jakobsson N., Sprei F. (2017). On the distribution of individual daily driving distances. Transp. Res. Part B Methodol..

[bib104] Raj T., Wang A.A., Monroe C.W., Howey D.A. (2020). Investigation of path‐dependent degradation in lithium‐ion batteries. Batteries & Supercaps.

[bib105] Rallo H., Benveniste G., Gestoso I., Amante B. (2020). Economic analysis of the disassembling activities to the reuse of electric vehicles Li-ion batteries. Resour. Conserv. Recycl..

[bib106] Rezvanizaniani S.M., Liu Z., Chen Y., Lee J. (2014). Review and recent advances in battery health monitoring and prognostics technologies for electric vehicle (EV) safety and mobility. J. Power Sources.

[bib107] Richardson R.R., Osborne M.A., Howey D.A. (2019). Battery health prediction under generalized conditions using a Gaussian process transition model. J. Energy Storage.

[bib108] Riezenman M.J. (1992). Electric vehicles. IEEE Spectr.

[bib109] Saxena S., Le Floch C., MacDonald J., Moura S. (2015). Quantifying EV battery end-of-life through analysis of travel needs with vehicle powertrain models. J. Power Sources.

[bib110] Schmalstieg J., Käbitz S., Ecker M., Sauer D.U. (2014). A holistic aging model for Li(NiMnCo)O2 based 18650 lithium-ion batteries. J. Power Sources.

[bib111] Shariff S.M., Iqbal D., Saad Alam M., Ahmad F. (2019). A state of the art review of electric vehicle to grid (V2G) technology. IOP Conf. Ser. Mater. Sci. Eng..

[bib112] Shi X., Pan J., Wang H., Cai H. (2019). Battery electric vehicles: what is the minimum range required?. Energy.

[bib113] Shinzaki S., Sadano H., Maruyama Y., Kempton W. (2015). Presented at the SAE 2015 World Congress & Exhibition.

[bib114] Simon B., Ziemann S., Weil M. (2015). Potential metal requirement of active materials in lithium-ion battery cells of electric vehicles and its impact on reserves: focus on Europe. Resour. Conserv. Recycl..

[bib115] Sopha B.M., Purnamasari D.M., Ma’mun S. (2022). Barriers and enablers of circular economy implementation for electric-vehicle batteries: from systematic literature review to conceptual framework. Sustainability.

[bib116] Stahel W.R. (2013). Policy for material efficiency—sustainable taxation as a departure from the throwaway society. Phil. Trans. R. Soc. A..

[bib117] Stan A.-I., Swierczynski M., Stroe D.-I., Teodorescu R., Andreasen S.J. (2014). 2014 International Conference on Optimization of Electrical and Electronic Equipment (OPTIM).

[bib118] Su L., Zhang J., Huang J., Ge H., Li Z., Xie F., Liaw B.Y. (2016). Path dependence of lithium ion cells aging under storage conditions. J. Power Sources.

[bib119] Suárez-Eiroa B., Fernández E., Méndez-Martínez G., Soto-Oñate D. (2019). Operational principles of circular economy for sustainable development: linking theory and practice. J. Clean. Prod..

[bib120] Uddin K., Dubarry M., Glick M.B. (2018). The viability of vehicle-to-grid operations from a battery technology and policy perspective. Energy Pol..

[bib121] UK Department for Transport, n.d. Vehicle licensing statistics data files [WWW Document]. GOV.UK. URL https://www.gov.uk/government/statistical-data-sets/vehicle-licensing-statistics-data-files (accessed 7.May.22).

[bib122] Vetter J., Novák P., Wagner M.R., Veit C., Möller K.-C., Besenhard J.O., Winter M., Wohlfahrt-Mehrens M., Vogler C., Hammouche A. (2005). Ageing mechanisms in lithium-ion batteries. J. Power Sources.

[bib123] Vidal C., Gross O., Gu R., Kollmeyer P., Emadi A. (2019). xEV Li-ion battery low-temperature effects—review. IEEE Trans. Veh. Technol..

[bib124] von Bülow F., Meisen T. (2023). A review on methods for state of health forecasting of lithium-ion batteries applicable in real-world operational conditions. J. Energy Storage.

[bib125] Wager G., Whale J., Braunl T. (2016). Driving electric vehicles at highway speeds: the effect of higher driving speeds on energy consumption and driving range for electric vehicles in Australia. Renew. Sustain. Energy Rev..

[bib126] Wang D., Coignard J., Zeng T., Zhang C., Saxena S. (2016). Quantifying electric vehicle battery degradation from driving vs. vehicle-to-grid services. J. Power Sources.

[bib127] Wang Q., Liu X., Du J., Kong F. (2016). Smart charging for electric vehicles: a survey from the algorithmic perspective. IEEE Commun. Surv. Tutorials.

[bib128] Wang Z., Ma J., Zhang L. (2017). State-of-Health estimation for lithium-ion batteries based on the multi-island genetic algorithm and the Gaussian process regression. IEEE Access.

[bib129] Wang K., Gao F., Zhu Y., Liu H., Qi C., Yang K., Jiao Q. (2018). Internal resistance and heat generation of soft package Li4Ti5O12 battery during charge and discharge. Energy.

[bib130] Wanitschke A., Hoffmann S. (2020). Are battery electric vehicles the future? An uncertainty comparison with hydrogen and combustion engines. Environ. Innov. Soc. Transit..

[bib131] Weil M., Ziemann S., Peters J., Pistoia G., Liaw B. (2018). Behaviour of Lithium-Ion Batteries in Electric Vehicles, Green Energy and Technology.

[bib132] Wen Yu, Zhang Wenjin, Lu Jiale (2015). 2015 Prognostics and System Health Management Conference (PHM).

[bib133] Weng C., Feng X., Sun J., Peng H. (2016). State-of-health monitoring of lithium-ion battery modules and packs via incremental capacity peak tracking. Appl. Energy.

[bib134] Wikner E., Thiringer T. (2018). Extending battery lifetime by avoiding high SOC. Appl. Sci..

[bib135] Woody M., Arbabzadeh M., Lewis G.M., Keoleian G.A., Stefanopoulou A. (2020). Strategies to limit degradation and maximize Li-ion battery service lifetime - critical review and guidance for stakeholders. J. Energy Storage.

[bib136] Yang L., Zhao L., Su X., Wang S. (2016). 2016 IEEE International Conference on Prognostics and Health Management (ICPHM).

[bib137] Yang D., Wang Y., Pan R., Chen R., Chen Z. (2017). A neural network based state-of-health estimation of lithium-ion battery in electric vehicles. Energy Proc..

[bib138] Yang Zhuo, Patil D., Fahimi B. (2017). 2017 IEEE Transportation Electrification Conference and Expo (ITEC).

[bib139] Yang F., Wang D., Zhao Y., Tsui K.-L., Bae S.J. (2018). A study of the relationship between coulombic efficiency and capacity degradation of commercial lithium-ion batteries. Energy.

[bib140] Yang S., Zhang C., Jiang J., Zhang W., Zhang L., Wang Y. (2021). Review on state-of-health of lithium-ion batteries: characterizations, estimations and applications. J. Clean. Prod..

[bib141] Yu B., Qiu H., Weng L., Huo K., Liu S., Liu H. (2020). A health indicator for the online lifetime estimation of an electric vehicle power Li-ion battery. WE!.

[bib142] Zhang C., Li K., Mcloone S., Yang Z. (2014). 2014 European Control Conference (ECC).

[bib143] Zhang J., Zhang L., Sun F., Wang Z. (2018). An overview on thermal safety issues of lithium-ion batteries for electric vehicle application. IEEE Access.

[bib144] Zhang Y., Wik T., Bergström J., Pecht M., Zou C. (2022). A machine learning-based framework for online prediction of battery ageing trajectory and lifetime using histogram data. J. Power Sources.

[bib145] Zhao Y., Pohl O., Bhatt A.I., Collis G.E., Mahon P.J., Rüther T., Hollenkamp A.F. (2021). A review on battery market trends, second-life reuse, and recycling. Sustainable Chemistry.

[bib146] Zhou Y., Huang M. (2018). On-board capacity estimation of lithium-ion batteries based on charge phase. J. Elect. Eng. Tech..

